# Expanding roles of cGAS-STING signaling in neuroinflammation

**DOI:** 10.1172/JCI204550

**Published:** 2026-06-01

**Authors:** Weixi Feng, Abulimiti Aikedan, Subhash C. Sinha, Li Gan

**Affiliations:** 1 Helen and Robert Appel Alzheimer Disease Research Institute, Feil Family Brain and Mind Research Institute, and; 2 Physiology, Biophysics and Systems Biology graduate program, Graduate School of Medical Sciences, Weill Cornell Medicine, New York, New York, USA.

## Abstract

The cyclic GMP-AMP synthase–stimulator of interferon genes (cGAS-STING) pathway is a central mediator of cytosolic DNA–induced innate immune responses, driving the production of type I IFNs and pro-inflammatory cytokines. Beyond its canonical role in cytosolic DNA sensing, increasing attention has been directed toward the noncanonical functions of cGAS and STING, particularly within the nucleus. Recent studies implicate dysregulated cGAS-STING signaling in neurodegenerative diseases and brain aging, with a prominent contribution to glial activation–associated neuroinflammation, a hallmark of many neurological disorders. In this Review, we first summarize the molecular mechanisms underlying the canonical cGAS-STING pathway in DNA sensing and innate immune activation. We then discuss emerging noncanonical roles of cGAS in chromatin organization and RNA metabolism, drawing on insights from evolutionary conservation and protein interactome analyses. Finally, we outline the involvement of cGAS-STING signaling in diverse aspects of brain function, including glial state regulation, neuronal homeostasis, blood-brain barrier integrity, and peripheral immune surveillance, highlighting their contributions to neuroinflammation and neuropathology. We also summarize current pharmacological inhibitors targeting cGAS and STING and discuss their therapeutic potential for modulating cGAS-STING signaling to manage brain disorders.

## Introduction

The sensing of nucleic acids is a central component of innate immunity, enabling host defense against infection while shaping inflammatory responses within the CNS. Pattern recognition receptors (PRRs) function as molecular sentries that detect both pathogen-derived nucleic acids and endogenous danger signals. Among these, DNA is an important signal of infection and inflammation (1–3). Several PRRs act as DNA sensors, including AIM2, TLR9, ZBP1, and cyclic GMP-AMP synthase (cGAS) (2–7). Unlike AIM2, which activates inflammasomes in response to DNA within the cytosol; TLR9, which senses DNA specifically in endosomes/lysosomes; and ZBP1, which recognizes the noncanonical left-handed Z-DNA structure, cGAS has the unique ability to directly and sequence-independently detect dsDNA in both the cytosol and nucleus (2, 4, 8–13). The dsDNA inside cells sensed by cGAS originates from diverse sources, ranging from foreign viral or bacterial DNA to endogenous self-DNA caused by mitochondrial damage or chromatin instability (14–20). Upon binding to dsDNA, cGAS activates the adaptor protein STING and elicits a strong IFN response (2, 14). cGAS is highly evolutionarily conserved from bacteria to mammals, underscoring its fundamental role in innate immunity (21).

Neuroinflammation is the immune response that occurs within the CNS and is the hallmark of multiple neurological diseases, including Alzheimer’s disease (AD), Huntington’s disease (HD), Parkinson’s disease (PD), and brain injuries (22–31). Neuroinflammation is characterized by activation of brain-resident cells, including microglia, astrocytes, and endothelial cells (ECs), and infiltration of peripheral immune cells, including monocytes, T cells, and B cells (28, 32–35). Sustained and dysregulated neuroinflammation has deleterious consequences on neuronal homeostasis and accelerates the progression of brain diseases.

Beyond its canonical role in antiviral immunity, the cGAS–stimulator of interferon genes (cGAS-STING) pathway has emerged as a key regulator of tumorigenesis, antitumor immunity, and neuroinflammation, thereby contributing to the pathogenesis of autoimmune diseases, neurodegenerative disorders, and aging (17, 36–43). In this Review, we focus on the role of cGAS-STING signaling in the brain, providing an integrated overview of its functions and therapeutic potential in neurodegenerative diseases.

## The molecular mechanisms of cGAS-STING signaling

### The function of cGAS-STING viewed through evolution.

**Figure 1:**
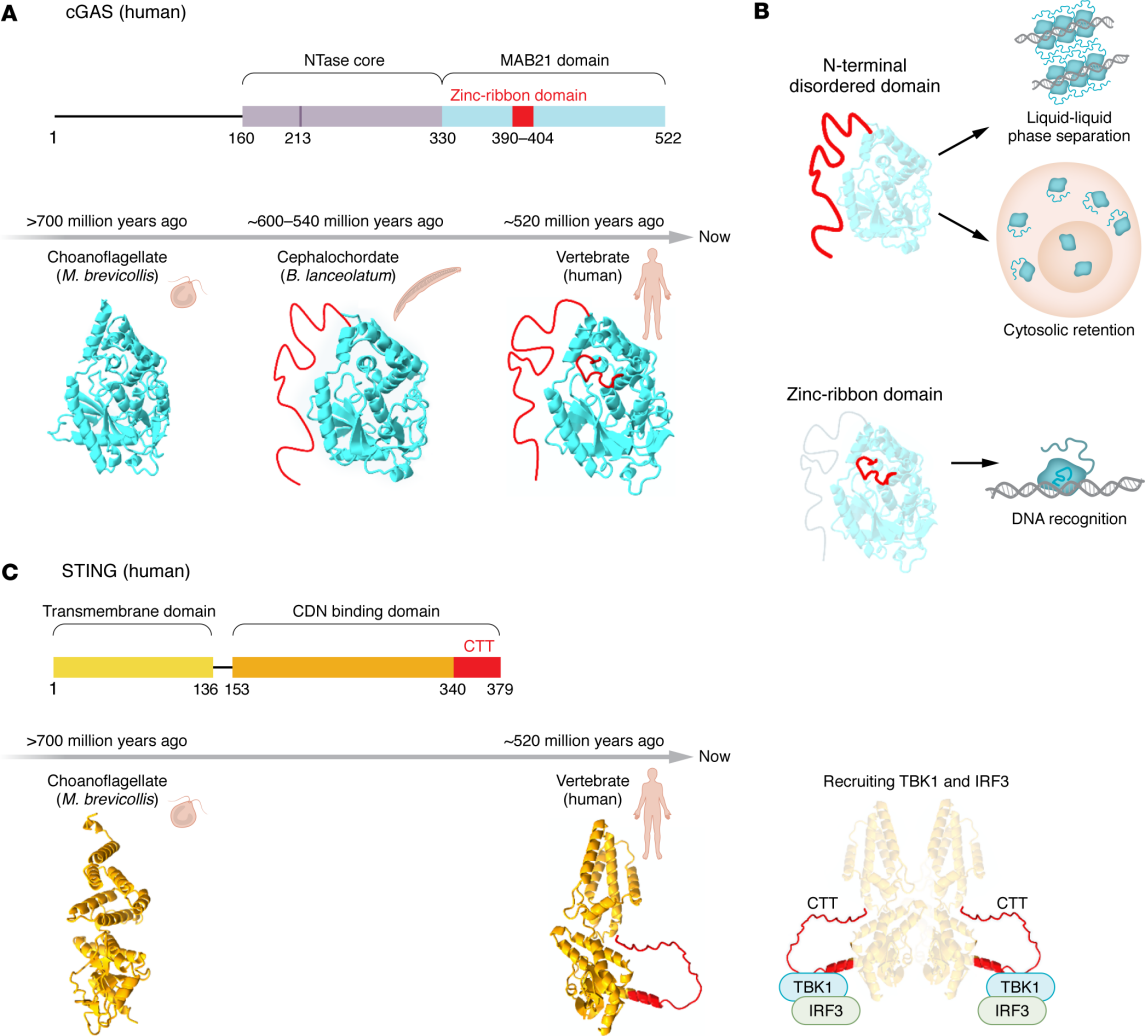
Evolutionary diversification of the cGAS-STING pathway. (**A**) Evolutionary timeline of cGAS across species. (**B**) Stepwise acquisition of cGAS functional domains. An N-terminal intrinsically disordered domain emerged in cephalochordates and promotes liquid-liquid phase separation and cytosolic retention, whereas the zinc ribbon domain arose later in vertebrates and is required for efficient DNA recognition and activation. (**C**) Evolution of STING signaling capacity. In vertebrates, STING acquired a C-terminal tail (CTT) that enables recruitment of TANK binding kinase 1 and interferon regulatory factor 3, driving robust type I interferon responses. This CTT-dependent signaling is absent in nonvertebrate STING homologs. The structures of cGAS and STING were downloaded from AlphaFold DB (170) and modified by ChimeraX (171). CDN, cyclic dinucleotide.

The cGAS-STING pathway represents an evolutionarily ancient innate immune system that predates the emergence of metazoans (44) (Figure 1A). cGAS has undergone substantial structural diversification during evolution. The cGAS protein encompasses a Mab21 catalytic domain at its C-terminus, which is highly conserved (45–48). In contrast, the N-terminal intrinsically disordered region (IDR), which contributes to the binding stability of dsDNA, exhibits marked divergence (21) (Figure 1B). In human cGAS, the IDR promotes liquid-liquid phase separation (LLPS) with DNA, enhancing nonspecific DNA binding and catalytic activity (47–49). Notably, truncated vertebrate cGAS without the N-terminus shows enhanced accumulation in the nucleus (50, 51). These observations suggest that ancestral cGAS proteins may predominantly localize to and function within the nucleus. Further evidence for functional diversification comes from the zinc-ribbon domain located in the C-terminal region of vertebrate cGAS, which is essential for efficient DNA recognition and activation (52) (Figure 1B). Strikingly, the cGAS homolog from *Nematostella vectensis*, which lacks the zinc-ribbon domain, can synthesize 2′3′-cyclic GMP-AMP (cGAMP) but fails to respond to dsDNA in vitro (53), supporting the notion that DNA sensing may represent a later evolutionary adaptation.

STING is also deeply conserved across species (21), yet its signaling capacity has expanded over time. Vertebrate STING acquired a C-terminal tail that enables recruitment of TANK binding kinase 1 (TBK1) and interferon regulatory factor 3 (IRF3), leading to robust type I IFN (IFN-I) signaling, a feature absent in nonvertebrate STING homologs (21) (Figure 1C). Together, these findings indicate that the cGAS-STING pathway has evolved from an antimicrobial strategy into a specialized IFN-driven DNA-sensing pathway and that in humans it likely serves additional functions beyond canonical responses to cytosolic dsDNA.

### Cytosolic DNA sensing by cGAS-STING.

**Figure 2:**
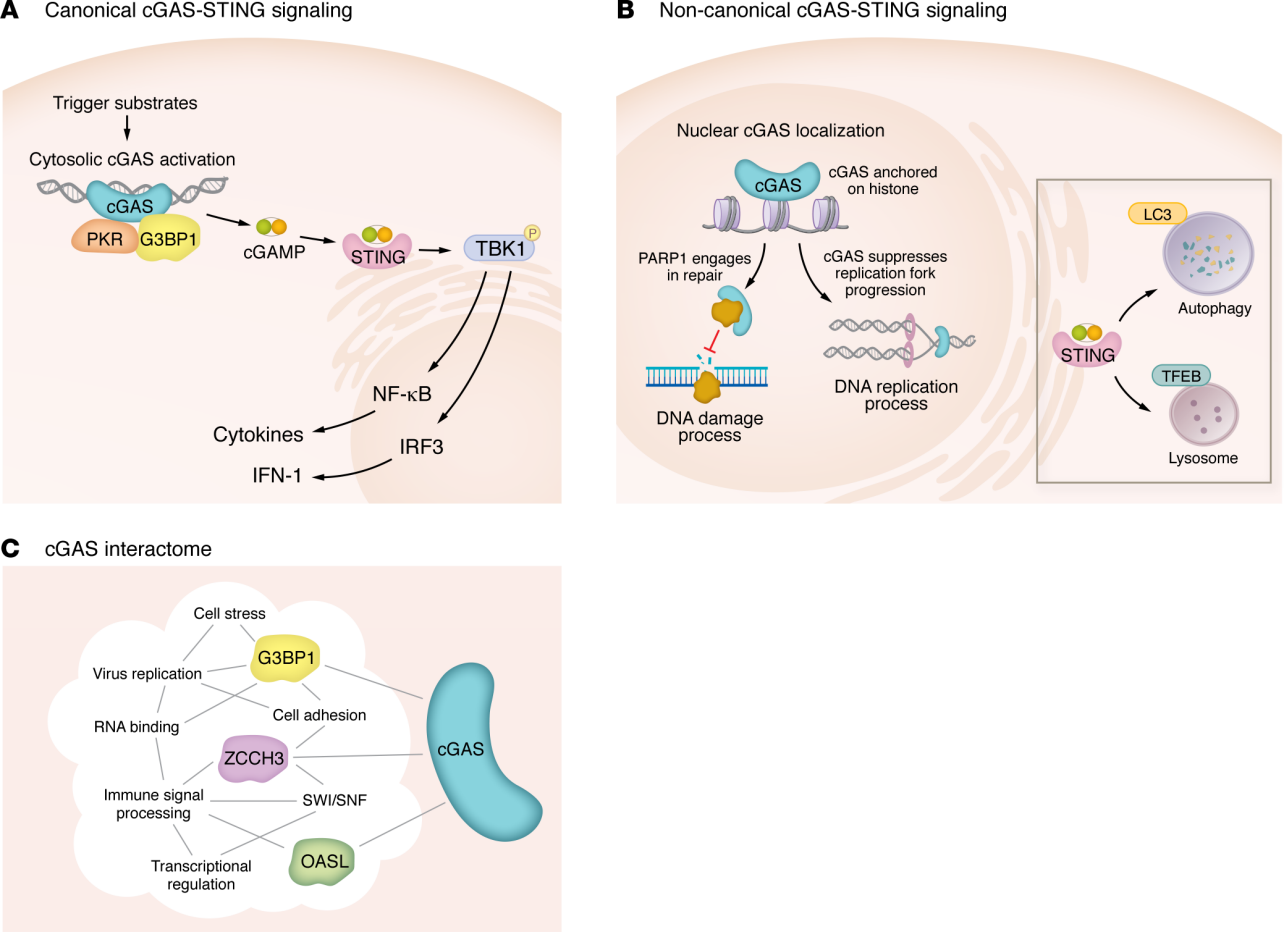
Canonical and noncanonical functions of the cGAS-STING pathway. (**A**) In the canonical signaling pathway, binding of dsDNA activates cGAS to synthesize cGAMP, which binds STING and drives its translocation from the ER to the ERGIC and Golgi. Activated STING recruits TBK1, leading to activation of IRF3 and NF-κB. (**B**) Among its noncanonical functions, STING activation can also lead to induction of autophagy and lysosome biogenesis via LC3- and TFEB-dependent pathways. In addition to its cytosolic role, cGAS localizes to the nucleus, where it is anchored to nucleosomes and mediates STING-independent functions, including inhibition of DNA damage repair through poly (ADP-ribose) polymerase 1 (PARP1) and suppression of replication fork progression. (**C**) Owing to its intrinsically disordered N-terminal domain, cGAS engages a broad network of interacting proteins, including RNA-binding proteins, transcriptional regulators, and the SWI/SNF chromatin-remodeling complex. These interactions suggest that cGAS may have additional, yet-to-be-defined noncanonical functions beyond innate immune signaling.

Upon binding to dsDNA, cGAS catalyzes the synthesis of cGAMP, from ATP and GTP (2, 14). Structural studies reveal that cGAS forms a 2:2 dimer with dsDNA (46). This results in formation of a ladder-like DNA-cGAS structure, in which cGAS dimers propagate linearly along longer DNA strands in a “head-to-head” orientation (54). Due to this ladder-like structure, longer dsDNA (>40 bp) binds more stably to cGAS and elicits greater production of cGAMP than shorter dsDNA (<40 bp) (54). The cGAMP binds to STING, an ER-resident adaptor protein, and promotes its translocation to the ER-Golgi intermediate compartment (ERGIC), mediated by COP-II complex and ARF GTPases (55). Activated STING recruits TBK1 via its C-terminal tail (56). STING is phosphorylated by TBK1 and subsequently recruits IRF3 (56). IRF3 is then phosphorylated by TBK1, dimerized, and translocated into the nucleus to drive the expression of IFN-I and interferon-stimulated genes (ISGs) (56–58) (Figure 2A).

Beyond the canonical IRF3-mediated IFN-I response, the cGAS-STING pathway engages multiple additional signaling programs that contribute to its diverse immune functions (55, 59–61) (Figure 2B). STING activates the NF-κB pathway through recruitment and activation of kinases, such as IκB kinase, which phosphorylate and promote degradation of the NF-κB inhibitor IκB (62, 63). This enables nuclear translocation of NF-κB and induction of pro-inflammatory cytokine expression, thereby amplifying inflammatory responses (63).

In parallel, cGAS-STING signaling directly intersects with autophagy and lysosomal pathways (55, 64) (Figure 2B). cGAS can recruit LC3 to micronuclei, while STING-enriched ERGICs provide a membrane source for LC3 lipidation, together facilitating autophagosome formation (55, 65). Activated STING also promotes TFEB dephosphorylation and nuclear translocation, driving the transcription of lysosomal and autophagy-related genes (64, 66, 67). These noncanonical signaling outputs underscore the functional scope of the cGAS-STING pathway beyond IFN-mediated inflammation and are consistent with its evolutionary diversification across species.

### Noncanonical nuclear cGAS-STING signaling.

cGAS is also present in the nucleus (51), where it is tightly tethered to nucleosomes rather than naked DNA, a configuration that prevents aberrant activation (43, 68–71). cGAS interacts with the acidic patch of histones H2A and H2B through its DNA-binding sites, but abnormal histone assembly leads to elevated cGAMP production by cGAS (43, 67, 68, 70, 72, 73) (Figure 2B). Another study showed that barrier-to-autointegration factor 1 can dynamically outcompete cGAS for DNA binding and restrict cGAS activity (74).

Despite these inhibitory mechanisms, nuclear cGAS can become active in the context of DNA damage following viral infection and produce cGAMP, eliciting a strong STING-dependent IFN-I response (8, 75). In addition, cGAS influences DNA damage repair (39, 43). Nuclear cGAS interacts with PARP1, disrupting formation of the PARP1-Timeless complex and thereby suppressing homologous recombination (43). In contrast with human cGAS, cGAS from the long-lived naked mole rat exhibits prolonged retention on chromatin and enhances DNA damage repair (39). DNA-bound cGAS also interacts with replication proteins, slows replication fork progression, and suppresses DNA damage sensitivity (76). Together, these findings suggest that nuclear cGAS regulates genome stability in a binding site– and cell state–dependent manner (Figure 2B).

The tight binding of nuclear cGAS to nucleosomes further suggests potential roles in shaping chromatin accessibility, higher-order chromatin architecture, and gene expression programs. A recent study profiling the cGAS interactome identified enrichment of cGAS-interacting intranuclear proteins within the SWItch/Sucrose Non-Fermentable (SWI/SNF) chromatin remodeling complex and the spliceosome complex, suggesting a role for nuclear cGAS in gene expression regulation (77). Further studies are needed to elucidate nuclear cGAS–interacting proteins and the functional consequences of nuclear cGAS on chromatin. In addition, it is important to understand how nuclear and cytosolic cGAS coordinate their functions. Human cGAS contains at least 2 nuclear localization sequence domains and 1 nuclear export signal domain (43, 78). Under homeostatic conditions, cGAS is present in both the nucleus and cytoplasm; however, DNA damage can shift its nucleocytoplasmic distribution. Nuclear DNA damage reduces cGAS Y215 phosphorylation and promotes its nuclear translocation in an importin-α–dependent manner (43). In contrast, cytoplasmic DNA damage triggers CRM1-dependent export of cGAS from the nucleus to the cytoplasm (78).

Cytosolic cGAS can also enter the nucleus following mitotic membrane breakdown, where its activity is restrained by nucleosome tethering and N-terminal hyperphosphorylation (72, 79, 80). This regulated nuclear entry may enable cGAS to monitor cell proliferation. As noted, nuclear cGAS can influence DNA damage repair through PARP1 (43), thereby affecting genome stability and cell proliferation. Importantly, during mitotic arrest, low levels of cGAS-dependent IRF3 phosphorylation gradually accumulate and trigger transcription-independent apoptosis in response to mitotic abnormalities (79).

Consistent with its nuclear function, STING is also located in the nucleus, particularly at the nuclear envelope (NE) (81). Upon herpes simplex virus type 1 or poly(I:C) stimulation, STING in the inner NE redistributes to the outer NE (82). Because the outer NE is continuous with the ER, NE-localized STING may represent a readily available pool of ER-associated STING that can participate in activation upon stimulation. STING has been reported to interact with the aryl hydrocarbon receptor (AHR), a ligand-activated transcription factor that regulates xenobiotic metabolism and immune homeostasis (83). Upon AHR ligand stimulation, STING accumulates at the NE, where it enhances AHR-mediated transcriptional activity (83). Notably, the NE is closely associated with heterochromatin organization, gene silencing, and RNA processing (84, 85). Given that a pool of STING localizes to the NE, it will be important to determine whether STING participates in these nuclear processes.

### Functions of cGAS revealed by interactome analyses.

Structurally, the N-terminal IDR of cGAS promotes LLPS (49), a property that enables extensive protein-protein interactions across diverse cell types and subcellular compartments. Consistent with this structural feature, recent cGAS interactome studies have revealed that cGAS associates not only with canonical components of innate immune signaling but also with proteins involved in transcriptional regulation and RNA processing (39, 77, 86, 87) (Figure 2C).

A particularly striking and recurrent finding across these analyses is the enrichment of RNA-binding proteins among cGAS interactors, likely driven by IDR-mediated phase separation. Notably, G3BP1, a core component of stress granules, directly binds the cGAS IDR and has been shown to prime cGAS for DNA binding (86, 88). Similarly, in the context of tauopathy, PQBP1 acts as an adaptor linking monomeric tau to cGAS (89). Given that both G3BP1 and PQBP1 are key regulators of RNA metabolism, these observations raise the possibility that cGAS may intersect with RNA-processing pathways, potentially by sequestering or scaffolding RNA-binding proteins within phase-separated assemblies. Exploring these noncanonical functions of cGAS may uncover mechanisms by which it contributes to aging and neurodegenerative disease in ways that parallel, yet remain distinct from, its classical role in inflammatory signaling.

## The cellular effectors of cGAS-STING in the CNS

**Figure 3:**
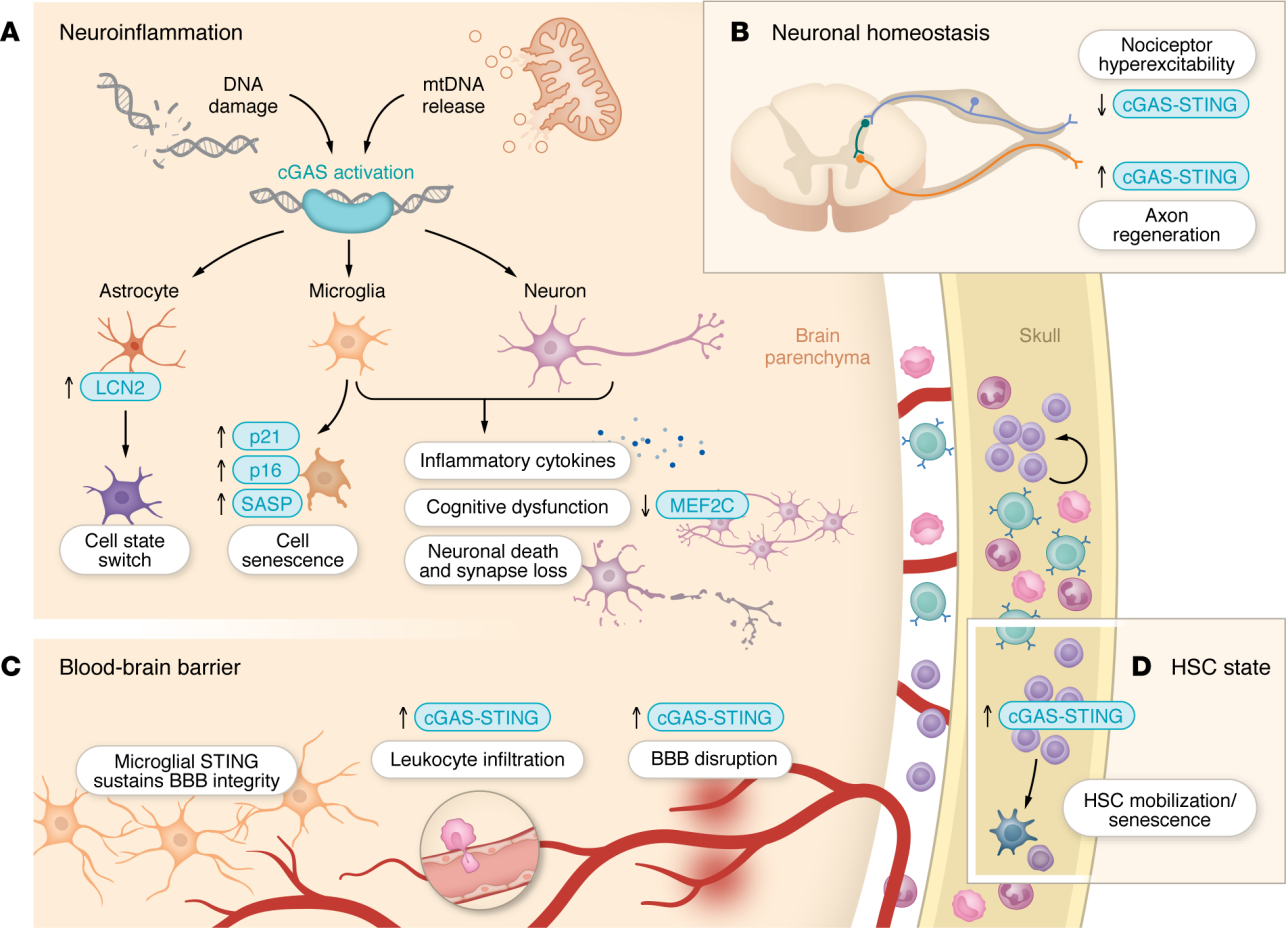
Cellular roles of the cGAS-STING pathway in the nervous system. (**A**) In neurodegenerative disease, pathological proteins activate cGAS-STING signaling in microglia, neurons, and astrocytes, driving chronic neuroinflammation, neuronal death, cellular senescence, and disease progression. (**B**) In the peripheral nervous system, neuronal STING signaling regulates nociceptor hyperexcitability and axonal regeneration after injury. (**C**) cGAS-STING signaling influences BBB integrity, with dysregulated activation promoting barrier disruption and leukocyte infiltration, while microglial STING activity supports barrier maintenance. (**D**) Suppression of cGAS-STING signaling maintains hematopoietic stem cell (HSC) quiescence, whereas aberrant STING activation disrupts homeostasis, promoting mobilization or senescence.

The CNS comprises specialized neurons and diverse glial populations that support neuronal function and homeostasis. Resident innate immune cells, microglia, continuously survey the brain parenchyma and maintain immune surveillance (90). Additional regulation is provided by the meningeal lymphatic system and skull bone marrow–derived immune cells (91–94), highlighting the complexity of CNS immunity. Here, we summarize the cellular and molecular effectors downstream of cGAS-STING activation in the CNS, focusing on how this pathway modulates neuroinflammation, neuronal homeostasis, and related processes (Figure 3).

### Glial states and inflammation.

Molecularly, sustained activation of the cGAS-STING/IFN-I pathway promotes activation of microglia and astrocytes across multiple brain disorders, leading to robust induction of ISGs and inflammatory cytokines (89, 95–97) (Figure 3A). These signaling molecules act in a paracrine manner on neighboring cells within the CNS, including neurons, thereby amplifying and propagating neuroinflammatory responses (98). Beyond cytokine production, cGAS-STING signaling has been implicated in microglial chemotaxis following genomic DNA damage and in driving astrocyte senescence in mouse models of PD (99, 100).

At the cellular scale, recent single-cell profiling studies (17, 38) further support a role for cGAS-STING signaling in shaping distinct glial states. Across models, this signaling axis consistently promotes pro-inflammatory glial subpopulations, in both microglia and astrocytes. Remarkably, these findings suggest that cGAS-STING not only amplifies inflammatory signaling but also contributes to the emergence of distinct glial subtypes and increases glial heterogeneity. Detailed disease-specific mechanisms are discussed below in the section on cGAS-STING as a therapeutic target in neurodegenerative diseases.

### Neuronal homeostasis and neuron-glia communication.

Although primarily expressed in innate immune cells, STING signaling also regulates neuronal inflammation, excitability, and regeneration (101–103). Neuronal STING restricts viral replication in *Drosophila* and promotes axonal regeneration in retinal ganglion cells or dorsal root ganglion neurons in mammals, while its loss leads to nociceptor hyperexcitability through dysregulated IFN-I signaling (101–103). These findings highlight a role for STING in maintaining neuronal function (Figure 3B). Whether similar roles extend to other neuronal subtypes, such as hippocampal neurons, remains unexplored.

Beyond cytosolic signaling, neurons experience high levels of activity-induced DNA damage, which is further exacerbated in neurodegenerative disease (104, 105). Given the presence of chromatin-bound cGAS and its reported involvement in DNA damage responses, it is intriguing to consider whether the noncanonical nuclear functions of cGAS contribute to the maintenance of neuronal genome stability under both physiological and pathological conditions. Defining how nuclear cGAS operates in postmitotic neurons may provide important insight into neuronal vulnerability during aging and neurodegeneration.

Beyond cell-autonomous functions, neurons can activate microglial cGAS-STING signaling through intercellular transfer of nucleic acid cargo, including micronuclei, mtDNA, and cGAMP (106–108), inducing inflammatory responses and reshaping microglial states. Notably, mitochondria themselves can be transferred between neurons and neighboring cells via extracellular vesicles or tunneling nanotubes (109, 110), suggesting that mtDNA transfer may represent a broader mechanism of neuron-glia communication. Understanding how cGAS-STING signaling engages these transfer processes will be important for linking neuronal stress to microglial activation in brain disorders.

### Blood-brain barrier integrity.

The blood-brain barrier (BBB), formed by ECs together with astrocytes and pericytes, restricts immune cell infiltration into the brain (111–113). cGAS-STING signaling is active in ECs, where damaged mtDNA serves as a major trigger (114). Across models, including irradiation, infection, and intracerebral hemorrhage, endothelial cGAS-STING activation induces IFN-I signaling and pyroptotic pathways, leading to BBB disruption and increased leukocyte infiltration into the brain (115–117) (Figure 3C).

Notably, the role of STING regarding BBB integrity appears to be context dependent. Although STING deficiency reduces cellular senescence, it paradoxically accelerates cognitive and motor decline, potentially because while endothelial activation is associated with BBB dysfunction, STING signaling in microglia seems to play a protective role by preserving hippocampal BBB integrity during aging (118). Interestingly, despite broadly suppressing canonical ISG expression, STING deficiency in aged mice is accompanied by upregulation of other disease-associated pathways, along with increased levels of the DNA damage marker phosphorylated histone H2A.X (γH2A.X) (118). These findings highlight that STING is engaged in divergent downstream pathways, underscoring the need to define STING signaling across different cell types and contexts.

### Peripheral immune cell–mediated brain surveillance.

Recent studies of the meningeal lymphatic system and skull bone marrow reveal that peripheral immune cells continuously survey the brain and infiltrate the parenchyma in both homeostasis and disease (33, 91, 93, 94, 119–122). Hematopoietic stem cells (HSCs) in the skull bone marrow contribute to cerebrospinal fluid (CSF) immune populations and maintain quiescence by suppressing cGAS-STING signaling, thereby avoiding IFN-I–mediated exhaustion (93, 94, 123) (Figure 3D). Conversely, activation of STING or stimulation with IFN-I leads to HSC mobilization or cellular senescence, respectively (124, 125). Given that the skull bone marrow is directly exposed to CSF and brain-derived signals (120, 126, 127), it remains unclear whether HSCs in this niche undergo similar exhaustion and influence immune surveillance of the brain in neurodegeneration and aging (17, 38).

STING signaling may also regulate immune cell infiltration in a context-dependent manner. Loss of STING in bone marrow–derived cells reduces immune infiltration and is neuroprotective in brain injury models (128, 129). In contrast, systemic STING activation can reduce T cell infiltration into the CNS by inducing Treg responses (130). STING activation also promotes regulatory B cell responses and may exert protective effects in neuroinflammation (131–133). These findings highlight distinct roles of cGAS-STING signaling regarding innate and adaptive immune compartments and suggest that it exerts both pro- and antiinflammatory effects in peripheral immune cells, though its cell type–specific functions and complex interactions with neuroglia remain incompletely defined (Figure 3D).

## cGAS-STING as a therapeutic target in neurodegenerative diseases

### Proteinopathies as triggers of cGAS-STING signaling.

**Figure 4:**
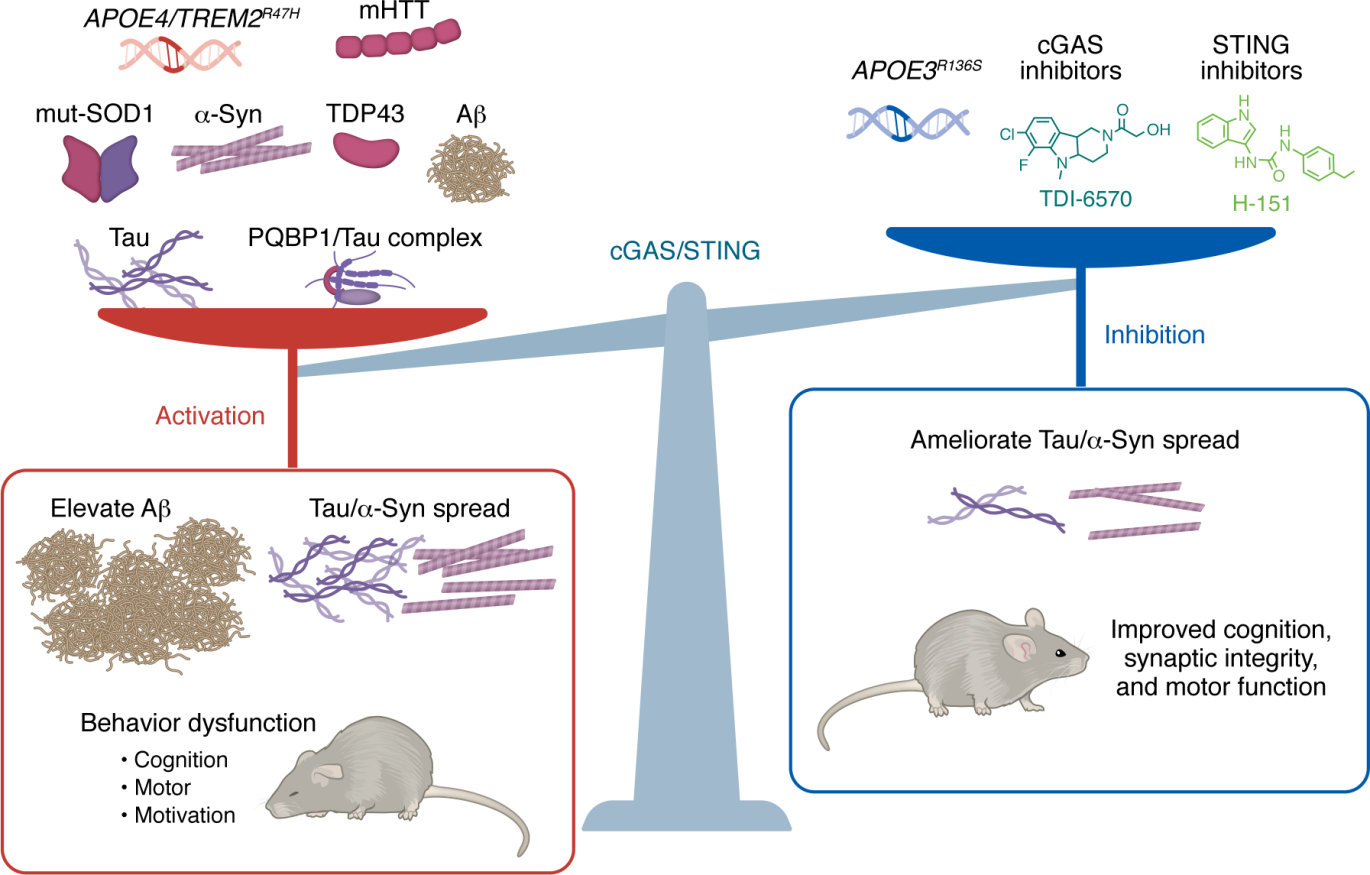
cGAS-STING signaling in neurodegenerative diseases. A broad spectrum of pathological protein aggregates including amyloid-β (Aβ), tau, α-synuclein (α-Syn), mutant SOD1, TDP-43, mutant huntingtin (mHTT), and the PQBP1-tau complex, as well as genetic risk factors such as *APOE4* and *TREM2^R47H^*, drives hyperactivation of the cGAS-STING pathway. Genetic ablation and functional studies indicate that excessive cGAS-STING signaling exacerbates protein pathology and promotes disease phenotypes, including cognitive, motor, and motivational dysfunction. In contrast, pharmacological inhibition of cGAS-STING (including RU.521 and TDI cGAS inhibitors and the STING inhibitor H151) or protective genetic variants (e.g., *APOE3^R136S^*) restore cellular homeostasis. Pathway suppression enhances beneficial effects such as reduced proteinopathy burden and increased resilience, evidenced by normalization of transcriptomic profiles and improved cognitive and motor performance.

Despite the diversity of pathogenic proteins implicated in neurodegeneration, a unifying principle is emerging: many proteinopathies ultimately generate cytosolic dsDNA capable of activating cGAS (Figures 3 and 4). Among the mechanisms, mtDNA leakage has been most consistently implicated and typically arises from mitochondrial stress or damage. Nuclear DNA damage represents an additional source of cytosolic dsDNA that can engage cGAS. Notably, an important exception to these DNA-dependent mechanisms is the tau-PQBP1 complex, which activates cGAS through a direct protein-protein interaction that bypasses the requirement for DNA (89).

Although cytosolic mtDNA release is widely recognized as a major trigger of cGAS activation across proteinopathies, the upstream molecular events linking pathogenic proteins to mtDNA release remain poorly defined in most neurodegenerative diseases. Overexpressed TDP-43 gains access to mitochondria via the TIM22 import machinery, leading to mitochondrial oxidative stress and opening of the mitochondrial permeability transition pore (mPTP) and voltage-dependent anion channel 1 (VDAC1), thereby allowing mtDNA to escape into the cytosol (37). Importantly, pharmacological inhibition of mPTP or VDAC1 effectively mitigates neurodegeneration in TDP-43 models. Tau fibrils have been detected within microglial mitochondria (17). Consistent with a mitochondrial source of innate immune activation, depletion of mtDNA attenuated the tau-induced IFN-I response. Furthermore, inhibition of Bcl-2–associated X protein (Bax), which promotes mitochondrial outer membrane permeabilization, reduced cGAS-driven microglial senescence, linking Bax-mediated mtDNA release to cGAS activation in tau-exposed microglia (40). Other pathogenic proteins, such as Aβ and mutant huntingtin, are also known to associate with mitochondria (17, 107, 134). It remains unclear whether these pathogenic proteins physically translocate into the mitochondrial matrix, which mitochondrial pores or channels mediate mtDNA release, and whether these processes are conserved across cell types. Moreover, mtDNA release mechanisms appear to be both cell type specific and trigger dependent. For example, inhibition of mPTP fails to reduce cytosolic mtDNA in a neuronal line harboring a distinct TDP-43 mutation, underscoring that different stress contexts may engage alternative mitochondrial pathways (135). This heterogeneity highlights the need for caution when extrapolating findings across disease models.

Regarding STING activation, accumulating evidence suggests that it does not strictly require canonical cGAS activation. Organelle stress, including ER stress, vesicular trafficking defects, and endolysosomal dysfunction, can induce STING overactivation and IRF3 phosphorylation, in some contexts independently of cGAS (95, 136, 137). Impaired STING degradation appears to be a common mechanism, leading to prolonged signaling (95, 137). Although few neurodegenerative models have been examined, Npc1 deficiency and C9orf72 loss of function support the relevance of these pathways (95, 138), suggesting that lysosomal defects, in addition to DNA leakage, may trigger STING activation independent of cGAS in neurodegenerative diseases. These findings raise the possibility that STING acts as a broader integrator of cellular stress signals beyond cytosolic DNA, contributing to IFN activation in neurodegeneration. In this context, cGAS ablation and sensitive cGAMP detection provide key approaches to distinguish cGAS-independent STING activation.

### cGAS-STING–driven maladaptive innate immune responses.

IFN-I signaling, beyond serving as a downstream molecular readout of cGAS-STING activation, has emerged as a key contributor of neurodegenerative pathology, particularly in AD. Recent studies demonstrate that sustained IFN-I signaling promotes microglial activation, synapse loss, plaque accumulation, and cognitive decline (139, 140). Consistently, elevated expression of ISGs, the downstream effectors of IFN-I signaling, together with pro-inflammatory cytokines, are common features across neurodegenerative mouse models, human patient tissues, and in vivo glial cells exposed to disease-related stressors.

Across diverse neurodegenerative conditions, accumulating evidence indicates that microglial cGAS-STING signaling acts as a major upstream regulator of IFN-I responses, contributing to gliosis and disease progression. Notably, mice expressing constitutively active STING develop α-synuclein pathology accompanied by robust neuroinflammation that ultimately leads to neurodegeneration, even in the absence of additional insults. Importantly, inflammatory changes precede neuronal loss in this model, including in juvenile mice (141), supporting a role for cGAS-STING as a proximate driver of pathology rather than a secondary consequence of neurodegeneration.

Microglial activation in response to abnormal protein accumulation is widely regarded as a double-edged sword (142). While activated microglia can engulf pathological aggregates and limit proteotoxic burden, excessive or chronic activation of microglia and astrocytes also represents a major source of neurotoxicity through mechanisms such as glutamate excitotoxicity, oxidative stress, and sustained release of pro-inflammatory cytokines (Figure 3). Intriguingly, genetic ablation or pharmacological inhibition of cGAS or STING selectively suppresses the neurotoxic components of gliosis while preserving beneficial functions in multiple neurodegenerative disease models. In models of AD, PD, and frontotemporal dementia, cGAS-STING inhibition markedly reduces IRF3- and NF-κB–driven inflammatory mediators, including ISGs and cytokines (37, 107, 135, 141, 143, 144). Remarkably, although the overall number of activated microglia is reduced, cGAS deletion simultaneously enhances Aβ clearance by increasing microglial recruitment to plaques in AD models (107). These findings suggest that targeting cGAS-STING can suppress toxic inflammatory programs while preserving, or even augmenting, phagocytic capacity (Figure 4).

This functional uncoupling of toxicity and clearance is further supported by disease-associated shifts in glial phenotypes (145). cGAS-STING inhibition reduces the neurotoxic A1 subtype of astrocytes in AD models (107) and promotes the neuroprotective A2 subtype in PD models (143). In addition, cGAS-STING signaling contributes to astrocyte senescence, and astrocyte-specific deletion of cGAS robustly attenuates neurotoxicity (100). Microglial state transitions are more complex and disease dependent. In PD models, cGAS promotes the expansion of neurotoxic microglial populations, an effect reversed by cGAS deletion (146). Although the disease-associated microglia (DAM) signature is reduced by cGAS deletion in PD (143), studies in tauopathy models reveal a critical distinction: cGAS selectively drives a distinct IFN-I–enriched microglial subpopulation that correlates with synapse loss and cognitive impairment while leaving the canonical DAM program largely unchanged (17). Consistent with this finding, an independent study identified a similar IFN-associated microglial population in a mouse model with aberrant cGAS activation, which closely resembled an IFN-responsive microglia subset associated with neuroinflammation observed in AD but was distinct from DAM (38). Similarly, the major AD risk alleles *APOE4* and *TREM2* R47H synergistically amplify cGAS-STING/IFN-I signaling, driving a senescence-like microglial state marked by mitochondrial stress, cell cycle arrest, and acquisition of a senescence-associated secretory phenotype (40).

The progressive spread of pathological tau is a central driver of neurodegeneration and cognitive decline, and emerging evidence identifies microglia as active facilitators of this process (31, 147–149). Pathogenic tau directly activates microglial NF-κB signaling, inducing a transcriptional program that alters tau handling and promotes the release of seed-competent tau species (150). Genetic inhibition of microglial NF-κB effectively traps tau intracellularly and markedly reduces extracellular tau seeding and in vivo propagation (150), establishing NF-κB as a key regulator of microglial tau export. The elevation of NF-κB activation could result from tau-induced activation of the cGAS-STING pathway in microglia (17), leading to sustained IFN-I responses that reinforce a maladaptive inflammatory state and further amplify tau spread. Consistent with this model, pharmacological inhibition of cGAS suppresses tau seeding and propagation while preserving core microglial functions (41) (Figure 4).

### Preserving neuronal resilience.

Neuron death is the proximal cause of functional decline in neurodegeneration. This can stem from two aspects: direct cytotoxicity from internalized protein aggregates or collateral damage induced by microglial hyperactivation, the latter evidenced by the death of bystander neurons lacking protein inclusions (89). While neuronal vulnerability can vary regionally across diseases, cGAS-STING inhibition consistently preserves synaptic integrity and rescues neuronal viability (100, 141, 144). This cellular rescue further translates into behavioral recovery. Behavioral improvements following cGAS-STING inhibition mirror the protection of specific neuronal populations: motor function is recovered in PD and amyotrophic lateral sclerosis models (96, 143, 151), while memory and cognitive performance are restored in AD-related pathologies (17, 107) (Figure 4). Intriguingly, beyond mere neuronal viability, cognitive resilience is also affected by cGAS hyperactivation. Single-nucleus sequencing reveals that microglial cGAS deletion reverses the downregulation of neuronal MEF2C in tauopathy (17). MEF2C is a transcription factor that orchestrates a gene network essential for axon morphology and neuronal function. This set of genes is upregulated in neurons from individuals who exhibit AD pathology yet retain cognitive function (17) (Figure 3). Thus, microglial cGAS/IFN-I signaling is associated with restraining the protective transcriptional program, dismantling the brain’s intrinsic resilience.

Recent work provides a compelling mechanistic link between cGAS-STING suppression and intrinsic neuronal resilience by demonstrating that pharmacological cGAS inhibition phenocopies the protective effects of the *APOE3* Christchurch (R136S) mutation in tauopathy models (41). Strikingly, cGAS inhibitor treatment not only preserves synapses and network function but also induces neuronal and microglial transcriptional signatures that closely mirror those conferred by the R136S resilience allele across cell types, providing direct pharmacological validation that suppression of cGAS-STING signaling is sufficient to recapitulate genetic protection against tau toxicity (Figure 4).

Given that microglia represent the dominant source of cGAS expression in the diseased brain, these neuroprotective effects are primarily attributed to non–cell-autonomous mechanisms. cGAS-STING inhibition improves the neuronal milieu by dampening chronic IFN signaling, reducing pro-inflammatory cytokine burden, and shifting glia away from senescent and neurotoxic states. Neuronal IFNAR1 likely serves as a key conduit linking microglial IFN-I release to neuronal dysfunction (17, 135). In addition, intercellular propagation mechanisms further amplify pathological signaling: cGAS-derived cGAMP can spread through gap junctions such as CX36, while PANX1 channels facilitate contact-independent dissemination of NF-κB–associated signals beyond the initial site of activation (135). Dissecting these microglia/neuron signaling axes will be essential for understanding how localized innate immune activation scales to circuit-level dysfunction.

At the same time, emerging evidence suggests that intrinsic neuronal cGAS activity may also contribute to disease progression in a cell-autonomous manner. Although neuronal cGAS expression is comparatively low under physiological conditions, functional cGAS-STING signaling has been observed in neurons subjected to pathological stress. Striatal neurons in HD models activate cGAS in response to mutant huntingtin–induced DNA damage (134), while Aβ42 exposure induces mtDNA release and cGAMP production in primary neurons (107). Moreover, STING inhibition reduces cell death in TDP-43–treated, induced pluripotent stem cell–derived motor neurons (96). Together with growing evidence for noncanonical, inflammation-independent functions of cGAS, these findings raise the possibility that neuronal cGAS contributes to neurodegeneration through distinct cell-autonomous mechanisms that remain to be fully defined.

### Considerations in current model systems.

While accumulating evidence supports a role for cGAS-STING signaling in neurodegeneration, several limitations of current experimental models warrant consideration, particularly when extrapolating to late-onset human disease. Many studies rely on overexpression systems (37) or strong pathway activation paradigms (141), which may amplify signaling beyond physiological levels. In addition, widely used models such as 5×FAD mice exhibit accelerated or early-onset phenotypes that do not fully capture the temporal progression of human disease (107). Injection- or toxin-based approaches can further introduce acute stress responses that differ from chronic neurodegeneration (141, 143).

Importantly, models incorporating human genetic risk factors, including *APOE4* and *TREM2* R47H (40, 41), may provide more disease-relevant contexts but remain underutilized in studying cGAS-STING signaling. Additional biological variables, such as sex differences, are also rarely examined despite their known impact on disease susceptibility and progression (152). Together, these considerations underscore the importance of validating key findings in physiologically and genetically relevant systems.

## Conclusions and future perspectives

cGAS-STING is a central regulator of neuroinflammation across brain disorders (17, 37, 40). Beyond microglia, this signaling axis is increasingly recognized as active in neurons, astrocytes, and ECs, where its potentially multifaceted functions remain poorly defined and warrant further investigation.

A major unresolved question concerns the function of nuclear cGAS. Phylogenetic analyses indicate that cGAS underwent structural modifications during vertebrate evolution, most notably the acquisition of a positively charged N-terminal tail (21). Experimental studies showing that deletion of this N-terminal region promotes nuclear localization of cGAS (50, 51) support the notion that nuclear cGAS may reflect an evolutionarily conserved function. Given the ability of cGAS to bind DNA and nucleosomes, it will be critical to define its precise subcellular localization, dynamics, and regulatory mechanisms within the nucleus. In particular, how nuclear cGAS influences chromatin organization, gene silencing, and transcriptional programs remains poorly understood. Moreover, because DNA damage is a common feature of neurodegenerative diseases and aging, whether nuclear cGAS participates in DNA damage sensing or repair in these contexts is an important question that warrants further investigation.

Neuroinflammation is typically characterized by persistent, low-grade inflammatory signaling (142), underscoring the importance of identifying the precise triggers and sustaining mechanisms of cGAS-STING activation in the CNS. Studies in AD models have implicated mtDNA leakage as a key activator of this pathway (17). However, whether additional sources of cytosolic DNA, or even non-DNA ligands, contribute to chronic cGAS-STING engagement remains unclear. This issue is particularly relevant in nonimmune cells such as neurons and ECs, where the mechanisms governing cGAS activation, signal amplification, and persistence are still largely undefined.

The growing recognition of cGAS-STING signaling as a driver of chronic neuroinflammation positions this pathway as an attractive, yet complex, therapeutic target for brain disorders. Sustained or inappropriate activation of cGAS-STING in the CNS is increasingly linked to neurodegeneration, cognitive decline, and aging. This highlights the need for therapeutic strategies that selectively attenuate pathological signaling while preserving essential antiviral functions. Pharmacological inhibition of cGAS or STING has shown encouraging results in preclinical models of neurodegenerative disease and brain injury, where suppression of IFN-I and downstream inflammatory cascades reduce neuronal loss and improve functional outcomes. Small-molecule cGAS inhibitors, STING antagonists, and modulators of cyclic dinucleotide metabolism represent the most direct approaches (Table 1).

While targeting the cGAS-STING pathway is of therapeutic interest, chronic inhibition may carry risks. Given its role in antiviral defense and tumor surveillance, sustained suppression could increase susceptibility to infection or impair immune function. A potential therapeutic strategy is partial and context-dependent modulation, as neurodegenerative conditions are often associated with elevated cGAS-STING activity and IFN-I signaling. To mitigate systemic effects, CNS-selective or temporally restricted approaches may be beneficial. These include brain-penetrant compounds and delivery strategies that limit peripheral exposure, as well as intervention during periods of pathway activation. Another potential strategy is to use PROTAC-based approaches that target downstream effectors of cGAS-STING signaling (153), rather than cGAS-STING directly, to improve specificity while minimizing systemic effects. Biomarkers will be important to guide these strategies. Although cGAMP and IFNs are short-lived (154, 155), downstream readouts, such as CSF IFN signatures (e.g., IP-10) and measures of glial activation, may serve as more practical indicators for patient selection and dose optimization. Further studies will be required to define safe and effective therapeutic windows. Over time, more refined strategies, including microglia-biased small molecules (156–159) or glia-targeted degraders (153), may further improve specificity and safety.

Beyond canonical IFN signaling, emerging noncanonical functions of cGAS and STING offer additional therapeutic opportunities. In neurons and glial cells, cGAS-STING signaling intersects with pathways regulating autophagy, mitochondrial quality control, cellular senescence, and DNA damage responses. Targeting specific downstream effectors or signaling branches may allow partial uncoupling of neurotoxic inflammation from beneficial immune surveillance. In particular, modulating nuclear cGAS functions or selectively interfering with chronic, low-level STING activation may prove advantageous in aging and slowly progressive neurodegenerative disorders.

Finally, effective therapeutic translation of cGAS-STING targeting strategies will require careful consideration of disease stage, cellular context, and signaling dynamics. Challenging the prevailing view that STING activation is uniformly deleterious in the aging CNS, recent work demonstrates that genetic loss of STING accelerates neurological decline (118), uncovering an unexpected homeostatic role for this pathway. While sustained or excessive cGAS-STING activation may contribute to pathology in established neurodegenerative disease, appropriately timed or cell type–restricted activation could be beneficial in early disease stages or following acute injury by facilitating the clearance of damaged cells or infectious agents. Accordingly, the development of biomarkers that report pathway activation state, cellular origin, and duration of signaling will be essential for patient stratification and the rational deployment of therapeutic interventions. In sum, a deeper understanding of cell type–specific signaling mechanisms, temporal regulation, and noncanonical functions of the cGAS-STING pathway will be critical for harnessing its therapeutic potential while preserving CNS homeostasis.

## Conflict of interest

LG and SCS are inventors of a published patent and filed a provisional patent application on human cGAS inhibitors, WO2023154962. LG is also a founder and SCS an advisor of Aeton Therapeutics, Inc.

## Figures and Tables

**Table 1:**
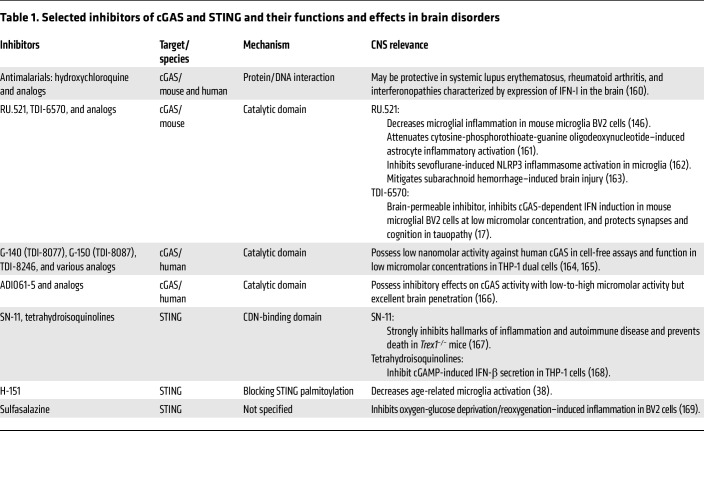
Selected inhibitors of cGAS and STING and their functions and effects in brain disorders

## Version 1 06/01/2026

Electronic publication
